# The Social Amoeba *Polysphondylium pallidum L*oses Encystation and Sporulation, but Can Still Erect Fruiting Bodies in the Absence of Cellulose

**DOI:** 10.1016/j.protis.2014.07.003

**Published:** 2014-09

**Authors:** Qingyou Du, Pauline Schaap

**Affiliations:** College of Life Sciences, University of Dundee, MSI/WTB/JBC complex, Dow Street, Dundee, DD15EH, UK

**Keywords:** Encystation, Amoebozoa, *Acanthamoeba* keratitis, cellulose synthase, cell wall biosynthesis, *Polysphondylium pallidum*.

## Abstract

Amoebas and other freely moving protists differentiate into walled cysts when exposed to stress. As cysts, amoeba pathogens are resistant to biocides, preventing treatment and eradication. Lack of gene modification procedures has left the mechanisms of encystation largely unexplored. Genetically tractable *Dictyostelium discoideum* amoebas require cellulose synthase for formation of multicellular fructifications with cellulose-rich stalk and spore cells. Amoebas of its distant relative *Polysphondylium pallidum (Ppal)*, can additionally encyst individually in response to stress. *Ppal* has two cellulose synthase genes, *DcsA* and *DcsB*, which we deleted individually and in combination. *Dcsa-* mutants formed fruiting bodies with normal stalks, but their spore and cyst walls lacked cellulose, which obliterated stress-resistance of spores and rendered cysts entirely non-viable. A *dcsa-/dcsb-* mutant made no walled spores, stalk cells or cysts, although simple fruiting structures were formed with a droplet of amoeboid cells resting on an sheathed column of decaying cells. *DcsB* is expressed in prestalk and stalk cells, while *DcsA* is additionally expressed in spores and cysts. We conclude that cellulose is essential for encystation and that cellulose synthase may be a suitable target for drugs to prevent encystation and render amoeba pathogens susceptible to conventional antibiotics.

## Introduction

Amoebas and many other freely moving protozoa differentiate into immobile dormant cysts when exposed to nutrient depletion or other forms of environmental stress. As cysts, the organisms can survive adverse conditions from months to years, and, in the case of pathogenic protozoa, resist the challenges of antibiotic treatment and immune clearance. This resilience is due to the fact that the cells are metabolically inactive and surrounded by an impermeable cell wall. In fungi, the polysaccharide chitin is the main structural component of the cell wall (Free 2013), but in chromalveolate algae and oomycetes, green algae, and amoebozoa, such as *Dictyostelium discoideum* and *Acanthamoeba castellani*, the structural component is cellulose (Blanton et al., 2000, Dudley et al., 2009, Fugelstad et al., 2009, Michel et al., 2010, Roberts et al., 2002).

In the social amoeba *Dictyostelium discoideum (Ddis)*, a single cellulose synthase gene is essential for the construction of multicellular fruiting bodies, by synthesizing a cellulose stalk tube and the cellulose-rich walls of individual stalk cells and spores (Blanton et al. 2000). Many *Dictyostelium* species, such as the genetic model *Polysphon*-*dylium pallidum (Ppal)*, can alternatively encyst as single cells. *Ppal* also constructs architecturally more complex fruiting structures than *D.discoideum* with multiple regular whorls of side branches.

For synthesis of the stalk tube, cellulose microfibrils are deposited at the exterior face of the plasma membrane of prestalk cells by single linear arrays of membrane-spanning cellulose synthases. While prestalk cells are maturing into stalk cells, the long linear arrays rearrange into multiple parallel rows for synthesis of the thicker fibrils of the stalk cell wall (Grimson et al. 1996). The spore wall consists of a cellulose layer sandwiched between two protein-rich layers. Spore coat proteins are presynthesized in Golgi-derived vesicles, which synchronously fuse with the plasma membrane at the onset of spore maturation. Cellulose deposition occurs somewhat later, starting at one pole of the spore and travelling towards the other pole. The spore wall cellulose is essential for proper deposition of the two proteinaceous layers of the spore coat (Zhang et al. 2001). Cellulose also makes up 28% of the *Ppal* cyst wall (Toama and Raper 1967), but cellulose synthases do not appear to form linear arrays in the plasmamembrane of encysting cells (Erdos and Hohl 1980).

*Acanthamoeba castellani* is an opportunistic pathogen that causes vision-destroying keratitis and lethal encephalitis, with cysts preventing effective treatment (Siddiqui et al. 2013). Cell wall biosynthesis is a major target for bacterial and fungal antibiotics and herbicides (Bush, 2012, McCormack and Perry, 2005, Wakabayashi and Böger, 2002). *Acanthamoeba* encystation was shown to be reduced by 85% by 0.48 mM of the herbicide 2,6-dichlorobenzonitrile, which inhibits plant cellulose synthesis (Dudley et al. 2007), and to 50% by incubation with small interfering RNAs against the *Acanthamoeba* cellulose synthase (Aqeel et al. 2013). Although not fully penetrant, these treatments show the potential importance of cellulose synthase for amoebozoan encystation. No gene knock-out strategies are as yet available for Amoebozoa outside Dictyostelia. The encysting dictyostelid *Ppal* therefore offers unique opportunities to identify and assess crucial roles of cellulose synthase genes in encystation. The differentiation of spores, stalk cells and cysts in Dictyostelia as well as encystation in *Acanthamoeba* all require cyclic AMP acting on PKA (Du et al., 2014, Kawabe et al., 2009, Reymond et al., 1995, Ritchie et al., 2008), which led to the working hypothesis that walled spore and stalk cells are evolutionary derived from cysts. *Ppal* can differentiate into all three cell types, allowing us to retrace how complexity in cell wall biosynthesis emerged.

A pilot study revealed the presence of two cellulose synthase genes in *Ppal*. In this work, we studied the expression patterns of both genes and abrogated the genes individually and together. Inspection of the null mutant phenotypes show both unique and overlapping roles for the cellulose synthases and an absolute requirement of cellulose synthesis for encystation and sporulation.

## Results

### Conservation of Cellulose Synthase Genes in Dictyostelia

The *D. discoideum* (*Ddis*) genome contains a single cellulose synthase gene, *DcsA*, and we first investigated whether *DcsA* is conserved throughout the dictyostelid phylogeny. The genomes of species representing the four major groups of Dictyostelia and the solitary amoebozoan *Acanthamoeba castellani* (*Acas*) (Clarke et al., 2013, Eichinger et al., 2005, Heidel et al., 2011, Sucgang et al., 2011) as well as all non-redundant sequences in Genbank were queried with the *Ddis* DcsA protein sequence, yielding single orthologues of *DcsA* in groups 1, 3 and 4 of Dictyostelia and an additional gene, *DcsB*, in *A. subglobosum* (*Asub*) and *Ppal*, which represent the two major clades of group 2. The dictyostelid cellulose synthase genes were more similar to bacterial and oomycete cellulose synthases than to the *Acas* cellulose synthase.

### Phenotype of a *Ppal dcsa-* Mutant

The group 2 species *Ppal* is the only encysting dictyostelid that is amenable to gene knockout procedures. To identify the respective roles of *DcsA* and *DcsB* in *Ppal*, we generated null mutants in either gene by transformation with a floxed neomycin cassette (Faix et al., 2004, Kawabe et al., 2009) flanked by ∼1 kb fragments of the *DcsA* or *DcsB* coding regions. Clones carrying gene knock-out (KOs) and random integration (RI) events were identified by two PCR reactions and Southern blot analysis (Supplementary Material Figs S1 and S2).

**Figure 1:**
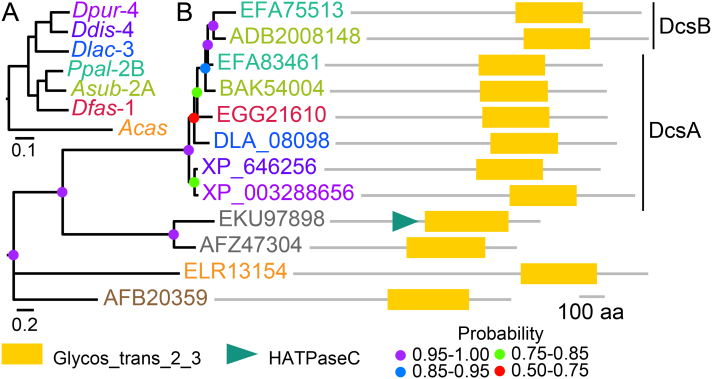
Phylogeny of dictyostelid cellulose synthases. **A**. Dicytostelid phylogeny. Genome-based phylogeny of group-representative *Dictyostelium* species and *Acanthamoeba castellani (Acas)* (Romeralo et al. 2013) with numbers referring to the relevant group or clade. *Dpur: D. purpureum, Ddis: D. discoideum, Dlac: D. lacteum, Ppal: Polysphondylium pallidum, Asub: Acytostelium subglobosum, Dfas: D. fasciculatum.***B**. Cellulose synthase phylogeny*.* Amoebozoan cellulose synthase genes and their closest homologs in other organisms were retrieved by BlastP search of Genbank and ongoing *D. lacteum* (http://sacgb.fli-leibniz.de and *A. subglobosum* (http://acytodb.biol.tsukuba.ac.jp) genome projects, using *Ddis* DcsA as bait. The regions containing the glycosyl transferase domain were aligned using Clustal Omega (Sievers et al. 2011) and subjected to phylogeny reconstruction by Bayesian inference (Ronquist and Huelsenbeck 2003). The phylogenetic tree is annotated with the functional domain architecture of the proteins, as analyzed with SMART (Schultz et al. 1998). The protein identifiers are color-coded according to species as in panel A, with grey further indicating the bacteria *Leptolyngbya sp.* (EKU97898) and *Cyanobacterium stanieri*, and tan the oomycete *Pythium iwayamai.* Bayesian posterior probabilities of tree nodes are indicated by colored dots.

**Figure 2:**
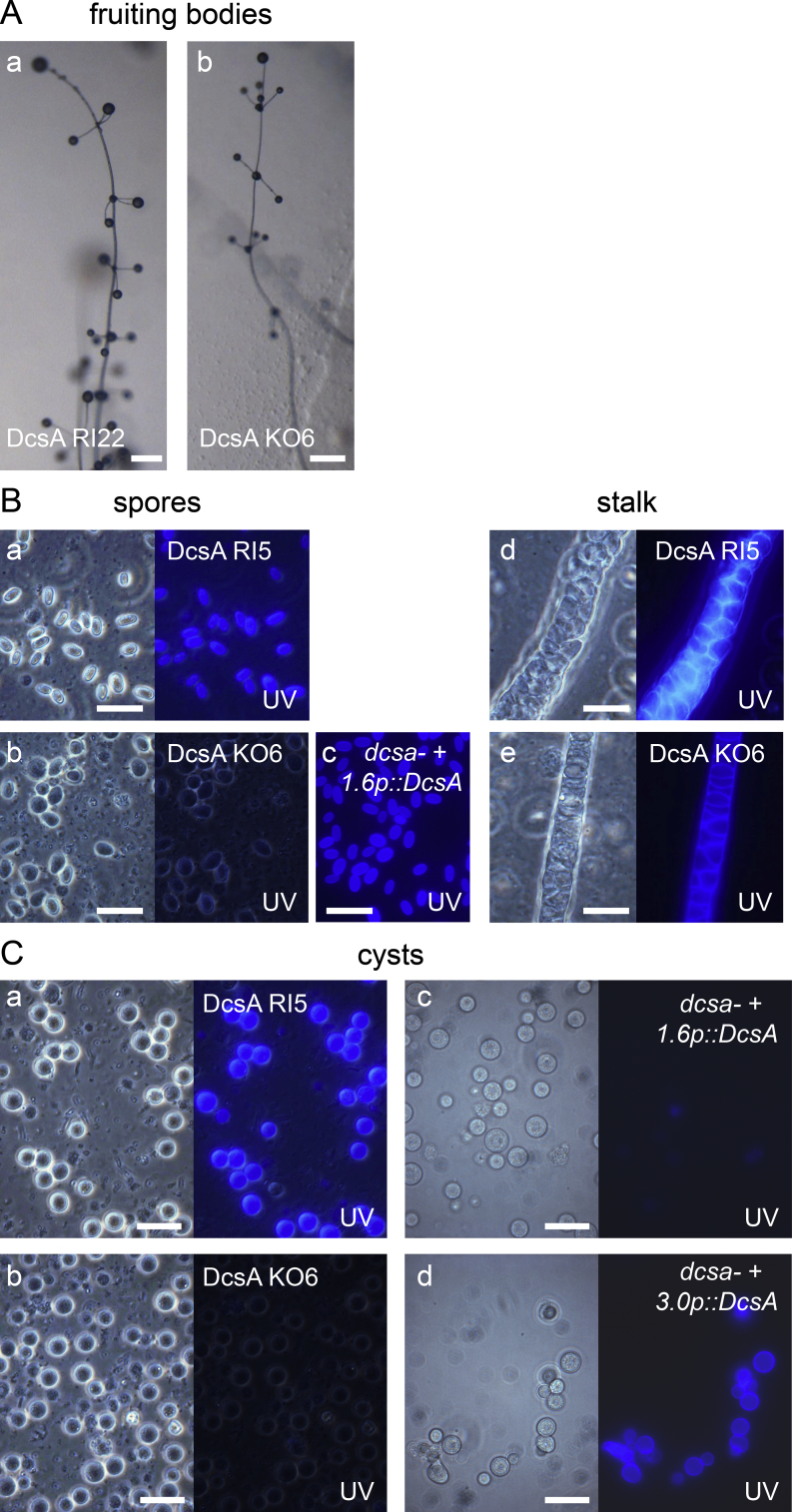
Phenotype of a *dcsa-* mutant. **A**. *DcsA* knockout (KO) and control random integrant (RI) cells were plated on PB agar and incubated until fruiting bodies had formed. Bar: 200 μm. **B**. Fruiting bodies of *DcsA* KO6 (a, d) and RI5 (b, e) cells, and of *dcsa-neo-* cells, transformed with the 1.6p::DcsA expression cassette (c) were transferred to 0.001% Calcofluor White on a slide glass. Spores and stalks were photographed under phase contrast (left panels), and under UV, combined with faint phase contrast illumination. Bar: 10 μm. **C**. *DcsA* KO6 (a) and RI5 cells (b) and *dcsa- cells* transformed with the 1.6::*DcsA* (c) or 3.0p::DcsA (d) cassettes were incubated in encystation medium. Calcofluor White was added to 0.001% after 4 days and cells were photographed. Bar: 10 μm.

Similar to control RI cells, *Ppal dcsa-* KO cells, formed fruiting bodies with normal stalks (Fig. 2A) that contained cellulose in their cell walls (Fig. 2B e). However, *dcsa-* spores, while still somewhat retaining their elliptical shape, contained little to no cellulose as evident by staining with the brightening agent Calcofluor White that interacts with cellulose (Fig. 2B b). Under submerged conditions, *Ppal* amoebas encyst individually when starved, and encystation is accelerated by high osmolarity. The *Ppal dcsa-* cells rounded off and lost their amoeboid shape when starved under these conditions, but unlike RI cells (Fig. 2C a), they did not produce the cellulose cell wall (Fig. 2C b).

To confirm that these phenotypes were caused by loss of *DcsA*, the neomycin cassette was deleted from *dcsa-* cells by transformation with *Cre* recombinase and the resulting *dcsa-neo-* cells were transformed with the *DcsA* coding region and 1.6 kb 5′intergenic sequence (Supplementary Material Fig. S1). This construct, *1.6p::DcsA*, restored cellulose deposition in spore walls (Fig. 2B c), but not in cyst walls (Fig. 2C c). We therefore prepared a second construct, *3.0p::DcsA*, with 3.0 kb intergenic sequence, which also restored cellulose synthesis in cysts (Fig. 2C d). These data show that DcsA is essential for cellulose synthesis in spores and cysts and that *DcsA* expression in either cell type is regulated by different promoter regions. Overall, the data show that *Ppal* DcsA is required for spore and cyst wall synthesis, but not stalk wall synthesis.

### Phenotypes of *dcsb-* and *dcsa-/dcsb-* Mutants

**Figure 3:**
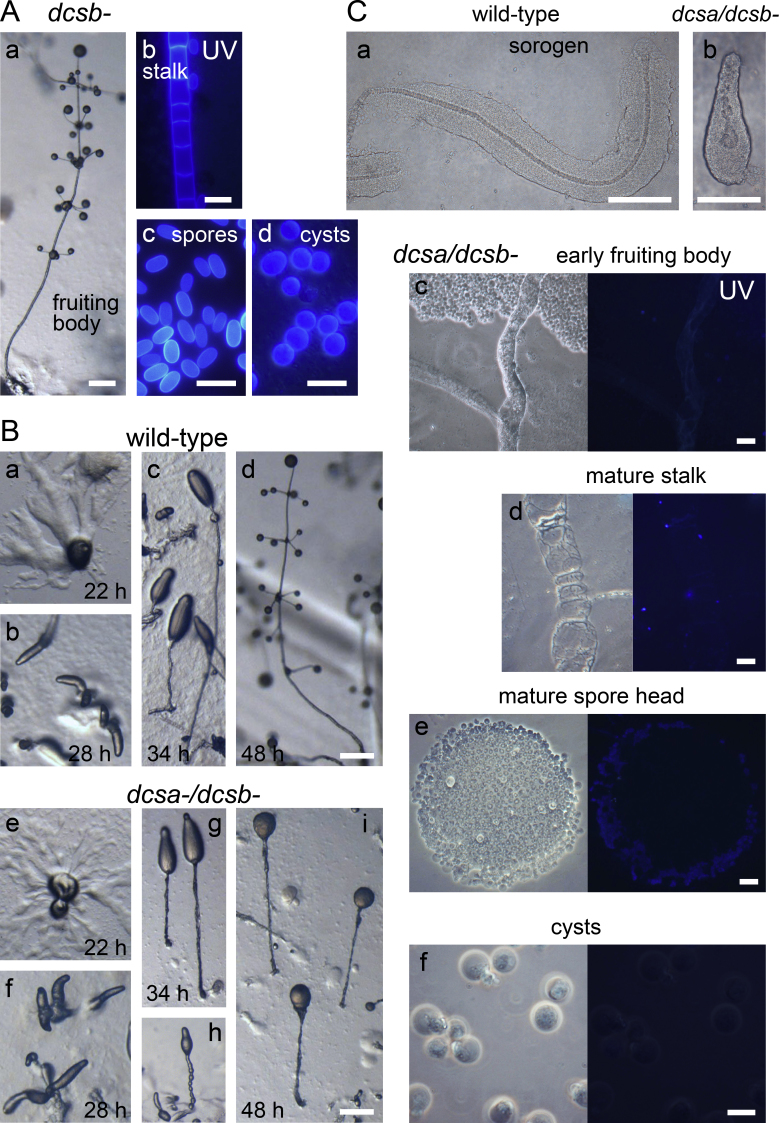
Phenotypes of *dcsb-* and *dcsa-/dcsb-* mutants. **A**. *Dcsb-* cells were developed to fruiting bodies on PB agar and to cysts in 400 mM sorbitol. Fruiting bodies were photographed *in situ* (bar: 200 μm), stalk cells, spores and cysts were stained with Calcofluor White and photographed under UV illumination. Bar: 10 μm. **B**. Wild-type *P. pallidum* and the *dcsa-/dcsb-* mutant were incubated on PB agar and photographed at the indicated time points. Bar: 200 μm. **C**. *dcsa-/dcsb-* and wild type sorogens and fruiting bodies were submerged *in situ* in 0.001% Calcofluor White, placed under a coverslip and photographed under phase contrast and UV illumination. Ca,b Bar: 100 μm; Cc,d,e,f Bar: 10 μm.

We next disrupted the *DcsB* gene, but surprisingly the *dcsb-* cells made normal cellulose-rich spore, stalk and cyst cell walls (Fig. 3A). This suggests that DcsB and DcsA have overlapping roles in stalk wall formation and to test this hypothesis, we generated a double *dcsa-/dcsb-* mutant. The phenotype of the *dcsa-/dcsb-* mutant was much more severe than that of the *dcsa-* mutant. The *dcsa-/dcsb-* mutant showed normal aggregation and formation of the primary sorogen (Fig. 3B e, f). The mutant did manage to erect stalked fruiting structures (Fig. 3B g-i), which often showed the pinched-off cell masses (Fig. 3B h), that give rise to the whorls of side branches in wild type *Ppal* (Fig. 3B c, d). These cell masses never developed into side-branches and the terminal fruiting structures usually consisted of a single mass of cells on top of an irregularly shaped stalk (Fig. 3B i). The cells at the interior of the “spore” mass were amoeboid and did not stain with Calcofluor White (Fig. 3C e). The *dcsa-/dcsb-* “spores” were also more isodiametric (length/diameter ratio 1.1 ± 0.1) than *dcsa-* spores (1.4 ± 0.2) and wild-type spores (1.8 ± 0.13), suggesting that DcsB still contributes somewhat to spore wall integrity and shape maintenance. The cells at the periphery of the *dcsa-/dcsb-* “spore” mass appeared to be lysed and showed weak staining throughout, which is probably caused by interaction of Calcofluor White with intracellular polysaccharides.

The stalk consisted of a fibrous sheath, that was initially filled with cell material (Fig. 3C c), but seemed empty in more mature structures (Fig. 3C d). There was none or very weak staining with Calcofluor White. Since wild-type stalk cells also die and leave little else behind than their walls, the *dcsa-/dcsb-* stalk cells may just be following their normal death programme. Even without a cellulose-rich tube the progression of stalk formation in *dcsa-/dcsb-* sorogens was similar as in wild-type sorogens, with newly formed stalk cells descending from the tip through the center of the cell mass to form the stalk (Fig. 3C a, b). Similar to *dcsa-* cells, the *dcsa-/dcsb-* cells also did not form cyst walls (Fig. 3C f).

The results indicate that DcsA is the primary enzyme for spore and cyst wall cellulose synthesis, and that DcsB has an overlapping role with DcsA in cellulose synthesis for the stalk tube and the walls of the stalk cells.

### Expression Patterns of *DcsA* and *DcsB*

**Figure 4:**
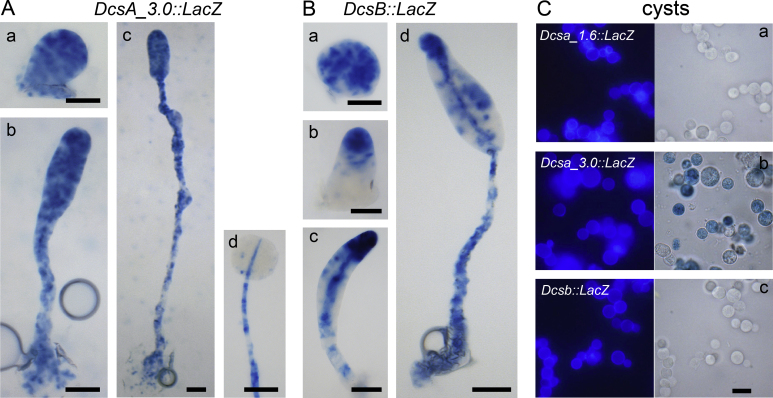
Expression patterns of *DcsA* and *DcsB*. **A/B**. Ppal wild-type cells transformed with the *DcsA3.0::LacZ* (**A**) and *DcsB::LacZ* (**B**) constructs were plated on nitrocellulose filters supported by PB agar. Emerging aggregates and early and late sorogens were fixed and stained with X-gal to visualize β-galactosidase activity. Bar: 50 μm. **C**. Cells transformed with *DcsA1.6::LacZ*, *DcsA3.0::LacZ* and *DcsB::LacZ* were incubated for two days in encystation medium. Cells were then fixed and stained with X-gal, counterstained with Calcofluor White to identify cysts, and photographed under UV and brightfield illumination. Bar: 10 μm.

We next investigated whether the apparent functional specialization of DcsA and DcsB is reflected by the expression pattern of their genes. The 1.6 and 3.0 kb *DcsA* promoter fragments and 2.7 kb *DcsB* promoter fragment (Supplementary Material Fig. S2) were fused to the *LacZ* reporter gene in plasmid pDd17 gal and transformed into *Ppal* wild-type cells. Developing structures were stained with X-gal to visualize activity of the cognate *LacZ* gene product, β-galactosidase. The *DcsA* 3.0 kb promoter activated *LacZ* expression in most cells in aggregates (Fig. 4A a) and in both early and late sorogens (Fig. 4A b, c), although X-gal staining tended to be somewhat more intense at the utmost tip and stalk. *DcsA* promoter activity disappeared completely from mature spores, but not from the stalks (Fig. 4A d). The 3.0 kb, but not the 1.6 kb DcsA promoter, was also active in encysting cells (Fig. 4C a, b).

Cells expressing *DcsB::LacZ* first appeared scattered throughout late aggregates (Fig. 4B a), but expression became rapidly restricted to the emerging tips (Fig. 4B b). In sorogens, *DcsB* promoter activity was high in the tip and stalk and some scattered cells throughout the sorogens (Fig. 4B c, d). There was no *DcsB* promoter activity in encysting cells (Fig. 4C c). The low or lacking expression of *DcsB* in prespore and cyst cells, respectively, is in good agreement with the fact that *DcsB* is not required for spore and cyst differentiation. The absence of *LacZ* expression from the *DcsA* 1.6 kb promoter in cysts also explains why expression of *DcsA* from the 1.6 kb fragment does not restore encystation. A more distal region contained in the 3.0 kb fragment is likely to mediate cyst-specific expression of *DcsA*.

### Viability of Spores and Cysts in Single and Double Cellulose Synthase Knockouts

**Figure 5:**
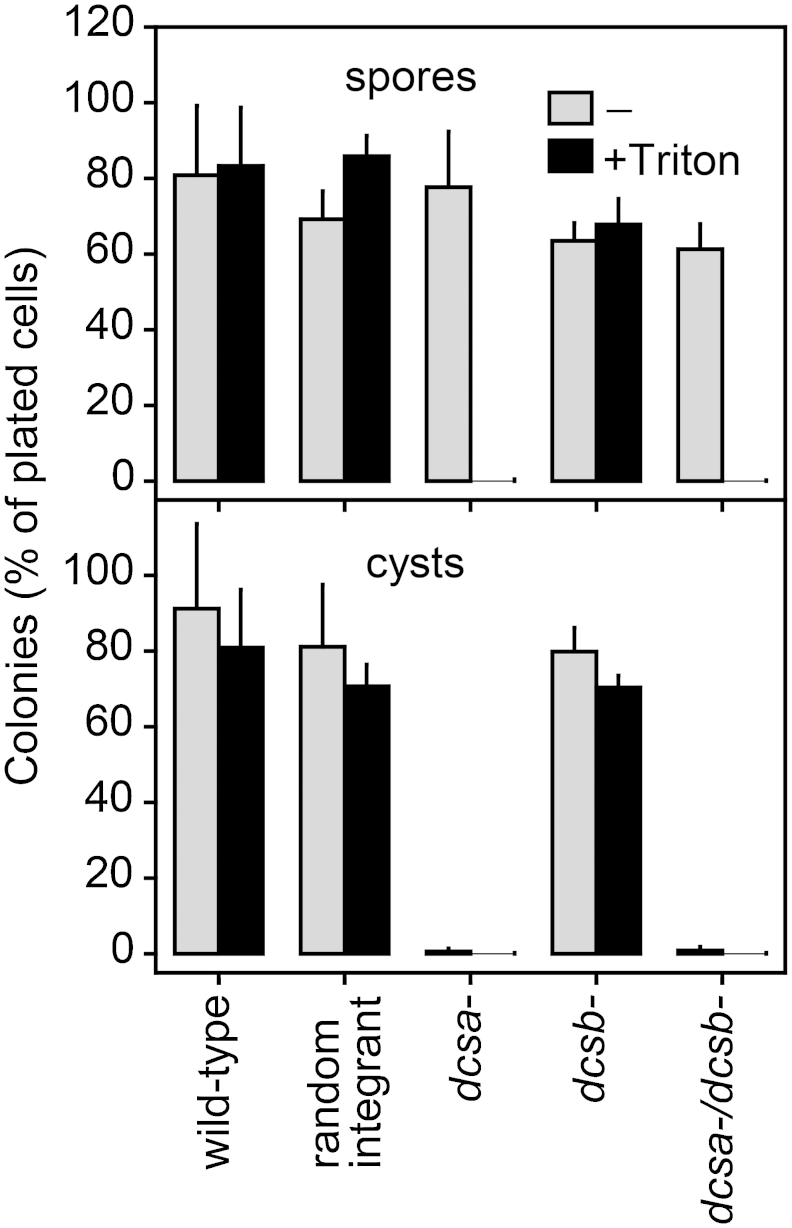
Spore and cyst viability of cellulose synthase null mutants. Wild-type, DcsA RI, *dcsa-*, *dcsb-* and *dcsa-/dcsb-* cells were harvested from mature spore heads or from 400 mM sorbitol after 4 days of incubation. Cells were counted and shaken for 10 min with and without 0.1% Triton-X100 before being plated at 500 cells/plate on *Klebsiella* lawns. After 3 days the emerging colonies were counted. The number of colonies as percentage of plated cells are shown, and the data are present means and SD of two experiments with duplicate plates for each variable.

We next assessed how loss of *DcsA* and/or *DcsB* affected spore and cyst viability. Spores were harvested from the sori of mature fruiting bodies, while cysts were obtained by incubating cells for 4 days in encystation medium. At this point, wild-type, *dcsb-* and *DcsA* RI cells had fully encysted. The cells were counted and shaken for 10 min in the presence and absence of 0.1% Triton-X100 before plating on *Klebsiella* lawnsʼ and after three days the emerging colonies were counted. About 70-80% of plated wild-type, *DcsA* RI and *dcsb-* spores formed colonies, regardless of detergent treatment (Fig. 5). The *dcsa-* and *dcsa-/dcsb-* spore equivalents still formed 80 and 60% colonies, respectively, in the absence of detergent treatment, but none after detergent treatment. Detergent treatment caused a small (∼10%) decrease in the number of colonies formed by wild-type, random integrant and *dcsb-* cysts (80-90% of plated cells). However, both the *dcsa-* and *dcsa-/dcsb-* cyst equivalents formed hardly any colonies in the absence of detergent treatment and none in its presence. Apparently, the *dcsa-* and *dcsa-/dcsb-* spore equivalents are viable, but not detergent resistant in the absence of cellulose, while the *dcsa-* and *dcsa-/dcsb-* cyst equivalents are entirely non-viable.

## Discussion

### Gene Duplication Followed by Functional Specialization of Group 2 Cellulose Synthases

Cellulose is a component of several structural features of *D. discoideum*, such as the slime sheath that surrounds the migrating slug, the walls of spore, stalk and basal disc cells and the supportive tube that surrounds the stalk cells. A single enzyme, DcsA, produces cellulose for all these features and its deletion prevents the formation of viable spores and of a stalk to lift the sorus from the substratum (Blanton et al., 2000, Zhang et al., 2001). Among the four dictyostelid taxon groups, the group 2 species *Ppal* and *Asub* have a second cellulose synthase gene, *DscB*. This gene most likely emerged by duplication of *DcsA*, since it is more similar to *DcsA* than to any gene outside Dictyostelia (Fig. 1). Our data indicate that in group 2 the two genes have started to acquire specialized functions. *DcsA* null mutants show severe defects in spore and cyst wall formation, but the stalk cell wall and stalk tube are still normally formed. While the walled cell types and multicellular structures of *dcsb-* mutants are not markedly different from those of wild-type *Ppal*, stalk formation becomes severely defective in a *dcsa-/dcsb-* mutant, indicating that DcsA and DcsB have an overlapping role in stalk formation. The expression patterns of the two genes reflect this partial specialization. *DcsB* is only expressed in prestalk and stalk cells, while *DcsA* is additionally expressed in prespore cells and from a separate distal promoter element in the cysts. The group 2 cellulose synthases seem to be on an evolutionary trajectory to perform specialized roles in cell wall synthesis.

### The *P. pallidum* Stalk is Rigid Without Cellulose

Unlike *Ddis dcsa-* sorogens, which entire fail to form a stalk (Blanton et al. 2000), the *Ppal dcsa-/dcsb-* sorogens still form a stalk tube-like structure with sufficient rigidity to keep an apical cell mass airborne (Fig. 3B h, i). While dictyostelid genomes do not contain chitin synthases (personal BLAST search), *D. discoideum* has two conserved extracellular matrix proteins, EcmA and EcmB, which consist of over 20 copies of a 24-amino-acid long repeat with 5 cysteine residues each. By forming extensive disulfide bridges these proteins contribute to the rigidity of the matrix, and EcmA was shown to enhance the tensile strength of the slime sheath (Morrison et al., 1994). At least three homologs of EcmA and EcmB are present in the *P. pallidum* genome (Genbank IDs: EFA80374, EFA79535 and EFA82732). It is plausible that the group 2 Polysphondylids with their habitually long thin stalks (Romeralo et al. 2013) have a larger abundance of these matrix proteins than *D.discoideum* with its shorter thicker stalks, and that this abundance allows the *dcsa-/dcsb-* mutant to form a cellulose-free stalk.

Similar to *Ddis dcsa-* prestalk cells (Blanton et al. 2000), the *Ppal dcsa-/dcsb-* prestalk cells still descend into the center of the cell mass attempting to form the stalk (Fig. 3C b). However, they never form a cell wall, and unlike *Ddis dcsa-* stalk cells, never vacuolate properly.

### Cellulose Synthesis is Essential for the Differentiation of Viable Cysts

The loss of *dcsa-* alone from *Ppal* is sufficient to prevent any viable cysts from being formed, highlighting an absolutely essential role for cellulose in cyst differentiation. While *Ppal* and most dictyostelids are harmless soil inhabitants, this is not the case for other Amoebozoa such as *Acanthamoeba* and *Balamuthia* sp. which can cause blinding keratitis and lethal amoebic encephalitis (Trabelsi et al., 2012, Visvesvara, 2010). Even the encysting dictyostelid *D. polycephalum* was shown to be responsible for a case of keratitis (Reddy et al. 2010). These infections resist antibiotic treatment, because the amoeba encyst in response to the perceived stress response. Eradication of the cysts requires months of painful treatment with a cocktail of antiseptics and antibiotics. The use of cellulose synthase as a target for weed killers (Wakabayashi and Böger 2002), shows that these enzymes can be effectively inhibited. However, existing plant cellulose synthase inhibitors do not always inhibit Amoebozoan cellulose synthesis (Kiedaisch et al. 2003) and may have unwanted side effects. By a developing an effective, non-toxic inhibitor for the amoebozoan cellulose synthase, and combining this compound with standard antibiotics, the treatment of amoeba keratitis could be fast, effective and painless.

## Conclusions

The encysting dictyostelid *P. pallidum* has two cellulose synthase genes. *DcsB* is expressed in prestalk and stalk cells and synthesizes stalk wall cellulose, together with *DcsA*. *DcsA* is additionally expressed in prespore cells and, from a more distal promoter element, in cysts. DcsA is required for production of spore and cyst wall cellulose and is essential for spore and cyst viability.

*P. pallidum* is the first genetically tractable model organism for systematic analysis of amoebozoan encystation, a process that renders amoebozoan pathogens impervious to immune attack and antibiotics. The essential role for cellulose synthase in cyst formation shown here, identifies this enzyme as a potential target for therapeutics to prevent encystation.

## Methods

**Cell culture:***Ppal* strain PN500 was grown in association with *Klebsiella aerogenes* at 22 °C on LP or 1/5^th^ SM agar. For multicellular development, cells were harvested from growth plates in 10 mM Na/K-phosphate, pH 6.5 (PB) and incubated at 10^6^ cells/cm^2^ on PB agar (1.5% agar in PB).

***DcsA*****and*****DcsB*****single and double knock-out mutants:** To obtain a *DcsA* knock-out plasmid, KO fragments *DcsA* I and II (Supplementary figure S1) were amplified from *Ppal* PN500 genomic DNA using primer pairs DcsAI5′/DcsAI3′ and DcsAII5′/DcsAII3′ (Supplementary Material Table S1), respectively, introducing XbaI/BglII sites on fragment I and HindIII/XhoI sites on fragment II. The fragments were sequentially inserted into the XbaI/BamHI and HindIII/XhoI digested plasmid pLox-NeoIII (Kawabe et al. 2012) yielding plasmid pDcsA_KO. Correct insertion was validated by DNA sequencing.

For a *DcsB* knock-out plasmid, *DcsB* KO fragments I and II (Supplementary Material Fig. S2) were similarly amplified with primer pairs DcsBI5′/DcsBI3′ and DcsBII5′/DcsBII3′ and inserted in pLox-NeoIII, yielding plasmid pDcsB_KO. The XbaI/XhoI inserts from the pDcsA_KO and pDcsB_KO plasmids were excised and 5 μg of either insert was transformed into 2.5 x 10^6^ *Ppal* cells together with 2 nanomoles of its flanking primers (Kuwayama et al. 2008). For transformation, *Ppal* cells were harvested from growth plates, incubated for 5 hours in HL5 at 2.5 x 10^6^ cells/ml and electroporated in ice-cold H-50 buffer with two pulses at a 5 s interval of 0.65 kV/25 μFd from a GenPulser2 (BioRad), followed by selection of transformants on autoclaved *K.aerogenes* at 300 μg/ml G418 (Kawabe et al. 1999). Knock-out clones were diagnosed by two PCR reactions and Southern blot analysis as illustrated in Supplementary Material Figures S1 and S2.

To generate a *dcsa-/dcsb-* double knock-out mutant, the floxed A6neo cassette was first removed from *dcsa-* KO6 by transformation with vector pA15NLS.Cre for transient expression of Cre recombinase (Faix et al. 2004). Transformed clones were replica-plated onto autoclaved *K. aerogenes* on LP agar plates with and without 300 μg/ml G418 for negative selection. The *dcsa-neo-* cells were subsequently transformed with the XbaI/XhoI insert from pDcsB_KO and screened for knock-out of *DcsB* as described above.

***DcsA*****expression constructs:** To express *DcsA* from its own promoter, a 4.59 kb genomic fragment including the *DcsA* coding region and 1.59 kb 5′ to the startcodon (Supplementary Material Fig. S1) was amplified by PCR using primer DcsAPro1_5′ and DcsA3′, which include XbaI and HindIII restriction sites, respectively (Supplementary Material Table S1). After XbaI/HindIII digestion, the fragment was ligated into similarly digested plasmid pExp5 (Meima et al. 2007), yielding plasmid 1.6p::DcsA, and validated by DNA sequencing. The plasmid was transformed into *dcsa-neo-* cells, but only partially restored the *dcsa-* phenotype. Therefore, a longer 6.19 kb fragment including 2.99 kb 5′ to the start ATG was amplified, using DcsAPro2_5′ (Supplementary Material Table S1) as the 5′primer, and inserted in pExp5, yielding 3.0p::DcsA.

***DcsA*****and*****DcsB*****promoter-LacZ constructs:** The 1.6 and 3 kb *DcsA* promoter fragments and a 2.7 kb *DcsB* promoter fragment (Supplementary Material Fig. S2) were amplified from *Ppal* genomic DNA using primer pairs DcsaPro1_5’/DcsApro3′, DcsaPro2_5′/DcsApro3′ and DcsB Pro5′/DcsBpro3′ (Supplementary Material Table S1), respectively. The 5′ and 3′ primers contain XbaI and BamHI restriction sites, respectively, which were used to insert the constructs into the BglII/XbaI digested vector pDdGal17 (Harwood and Drury 1990). This generated plasmids pDcsA_1.6::LacZ, pDcsA_3.0::LacZ and pDcsB::LacZ with the *LacZ* coding sequence fused at its 5′end to either of the three promoters. The plasmids were transformed into *Ppal* wild-type cells and β-galactosidase activity was visualized with X-gal as described previously (Kawabe et al. 2009). All plasmids and knock-out mutants that were generated in this study have been deposited in the *Dictyostelium* Stock Centre (http://dictybase.org/StockCenter/) or are available on request.

**Cyst and spore germination assay:** To obtain spores, *Ppal* wild-type cells and mutants were harvested from growth plates and incubated at 22 °C on PB agar for 4 days until mature fruiting bodies had fully formed. For cysts, cells were resuspended in encystation medium (PB with 400 mM sorbitol) and incubated for 4 days in the dark until wild-type cells had formed mature cysts. Spores and cysts, harvested from fruiting bodies and encystation medium, respectively, were resuspended in 80 mM sucrose in PB (Zhang et al. 2001) and counted. Triton-X100 (or an equivalent volume of water) was added to a concentration of 0.1%, cells were shaken for 10 min. and then diluted at least 100x in 80 mM sucrose for plating with *K.aerogenes* on 1/5^th^ SM agar plates at 500 cells per 15 cm plate. Colony numbers were counted after 3 days of culture at 22 °C.
