# Supplementary material for: The Social Amoeba Polysphondylium pallidum Loses Encystation and Sporulation, but Can Still Erect Fruiting Bodies in the Absence of Cellulose
Source: Protist. 2014 Sep;165(5):569–79. doi: 10.1016/j.protis.2014.07.003 (PMC4210663; doi:10.1016/j.protis.2014.07.003)
Supplement: Supplementary file 1 [file mmc1.doc]

**Supplementary table S1. Oligonucleotide primers used in this work**

| Name | Restriction site | Sequence |
| --- | --- | --- |
| DcsAI5’ | XbaI | 5’-GAT**TCTAGA**TGGAGAAGACCAAGGAGCG-3’ |
| DcsAI3’ | BglII | 5’-GAT**AGATCT**TGACGTCCCATCATCAACA-3’ |
| DcsAII5’ | HindIII | 5’-GAT**AAGCTT**TCGTACAGACTCCACAGT-3’ |
| DcsAII3’ | XhoI | 5’-GAT**CTCGAG**AATGACAAAGATGACAAT-3’ |
| DcsAneg3' |  | ACTCCATATTACGATGACCGAG |
| DcsAneg5' |  | CATGTACCGTCCACTGATCCGT |
| cas1 |  | GGGCAAATCTGTAATTTTCAG |
| DcsA_F |  | GGCAATACGTGGAATACAGACT |
| DcsBI5’ | XbaI | 5’-GAT**TCTAGA**TAACAGTAACCATACCGAG-3’ |
| DcsBI3’ | EcoRI | 5’-GAATTCCATGT**GGATCC**TCATTG-3’ |
| DcsBII5’ | HindIII | 5’-GAT**AAGCTT**CTCCATAGAGTCACTGAGA-3’ |
| DcsBII3’ | XhoI | 5’-GAT**CTCGAG**GAAATATAGGTTCCGGC-3’ |
| DcsBneg3' |  | GGTGTGATGAATCTAAGGTGAC |
| DcsBneg5' |  | GTCCACAAGATTGGTCCACC |
| DcsBpos3' |  | GGGCAAATCTGTAATTTTCAG |
| DcsBpos5' |  | TGTGACGACTGATACGACGTTG |
| DcsAPro1_5' | XbaI | GAT**TCTAGA**TTGGACATTAAGATGATTGG |
| DcsaPro2_5' | XbaI | GTAGTAGTTGTAG**TCTAGA**AGAAG |
| DcsA3’ | HindIII | GAT**AAGCTT**AGGCAATACGTGGAATACAG |
| DcsAPro3' | BamHI | GAT**GGATCC**TTTTGATTTACTAATAATAAAT |
| DcsBPro5' | XbaI | GAT**TCTAGA**TTTCAACATACTCTCACCAC |
| DcsBPro3' | BamHI | GAT**GGATCC**AATTATTATTTACCTATTAG |

**Supplementary figure S1. *DcsA* gene model, knock-out strategy and diagnosis**


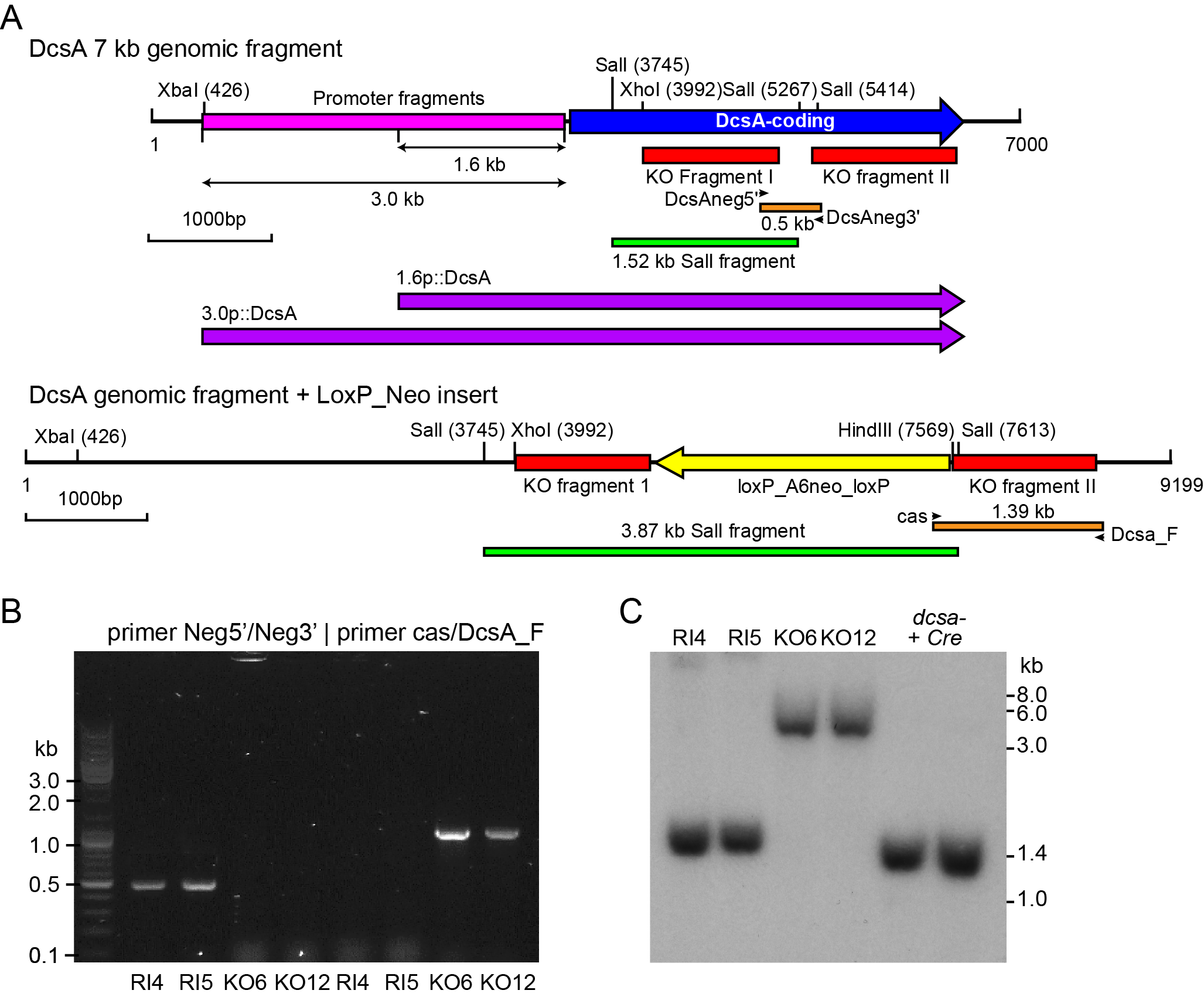


A. Model of a 7kb genomic fragment that harbours the *DcsA* gene before and after insertion of the loxP_A6Neo cassette by homologous recombination. The model also shows regions (in violet) that were amplified and cloned in pExp5 for expression of the *DcsA* coding sequence from a 1.6 kb and a 3.0 kb promoter fragment, as well as the same promoter fragments (in pink) that were fused to *LacZ* for histochemical analysis of promoter activity. The construction of the knock-out and expresssion plasmids is described in Methods.

B. To identify *DcsA* knock-outs (KOs), genomic DNAs from clones transformed with LoxP_A6neo flanked by *DcsA* KO fragments I and II were first subjected to PCR with primer pair DcsAneg5’ and DcsAneg3’ (Supplementary Table S1), which amplify a fragment of 0.5 kb in wild-type and random integrant (RI) clones. Putative KOs and some RIs from the first round were next tested with primers cas and DcsA_F (Table S1) that yield a 1.39 kb product after homologous recombination.

C. Genomic DNAs from two selected KO and RI clones were digested with SalI, size-fractionated on agarose and transferred to nylon membranes. The Southern transfer was probed with ^32^P-labeled KO fragment 1. The insertion of the LoxP_A6Neo cassette in *DcsA* KOs results in an increase of the SalI fragment size from 1.5 to 3.9 kb, as is evident when comparing RI4 and RI5 with KO6 and KO12. To generate a *dcsa-* strain that could be used for either expression of DcsA or sequential knock-out of DcsB, *DcsA* KO6 was transformed with vector pA15NLS.Cre for transient expression of Cre recombinase. Genomic DNAs of two clones that had lost G418 resistance were SalI digested and probed alongside the *DcsA* KO and RI clones. Both clones show reduction of the SalI band from 3.9 to 1.4 kb that results from loss of the LoxP-A6neo cassette.

**Supplementary figure S2. *DcsB* gene model, knock-out strategy and diagnosis of *dcsb-* and *dcsa-dcsb-* mutants**


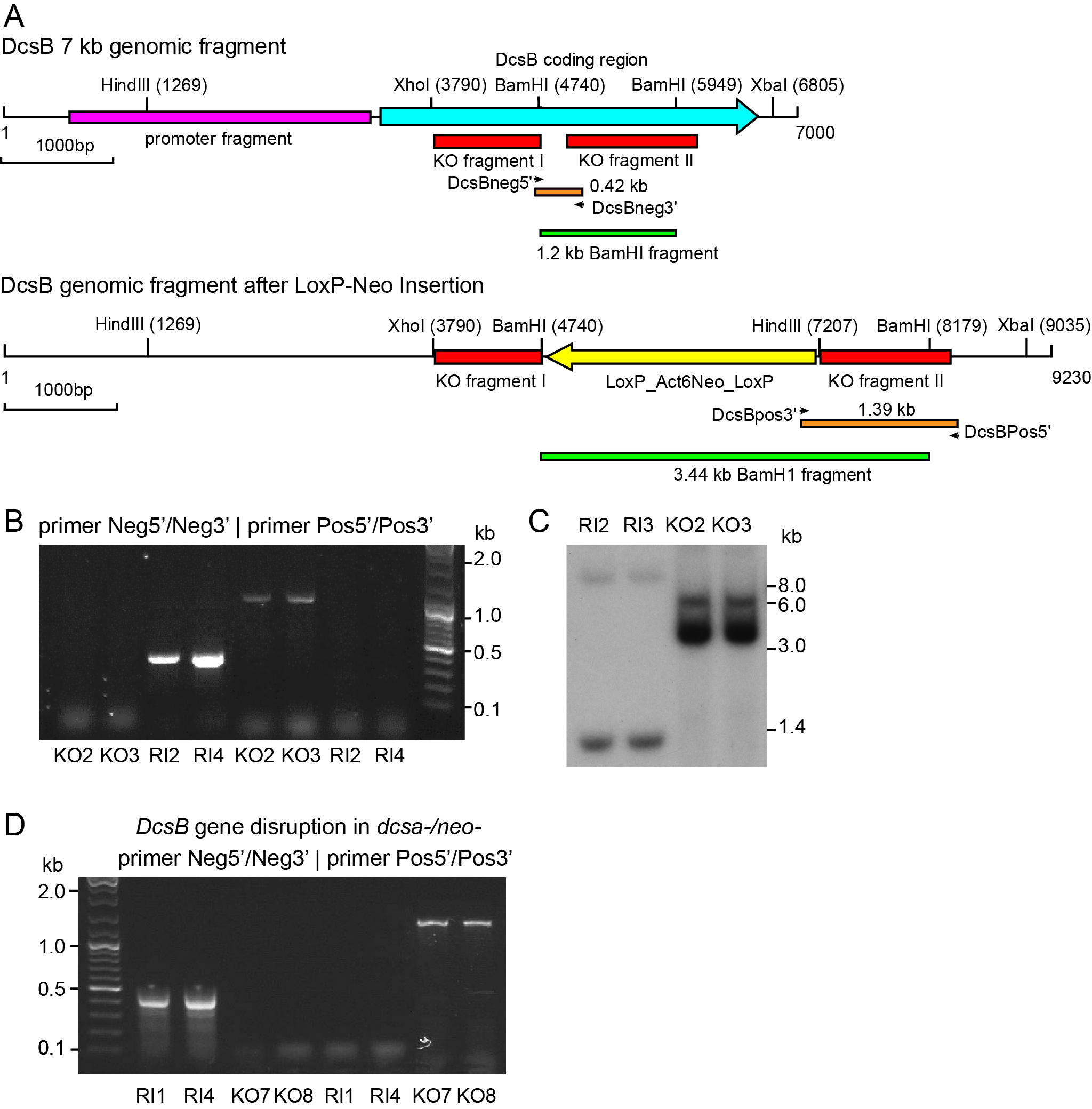


A. Model of a 7kb genomic fragment that harbours *DcsB* gene before and after insertion of the loxP_A6Neo cassette (see Methods). The promoter fragment that was fused to *LacZ* (see figure 4, main text) is shown in pink.

B. To identify *DcsB* KOs, genomic DNAs from G418 resistant clones of cells transformed with pDcsB_KO were first subjected to PCR with primer pair DcsBneg5’ and DcsBneg3’ (Supplementary Table S1), which amplify a 0.4 kb fragment in RI clones. Putative KOs and RIs were next tested with primers DcsBpos5’ and DcsBpos3’ (Table S1), which yield a 1.4 kb product in KO clones.

C. Genomic DNAs from two selected KO and RI clones were digested with BamHI and Southern transfers were probed with a ^32^PdATP-labeled *DcsB* KO fragment II. The insertion of LoxP_A6neo after homologous recombination results in an increase of the BamHI fragment size from 1.2 to 3.4 kb. The minor ~ 6kb band in the KO samples is probably caused by incomplete BamHI digestion.

D. Genomic DNAs from G418 resistant clones of *dcsa-/neo-* cells, transformed with pDcsB_KO were subject to PCR with the same primer pairs as used in panel B. Two *dcsa-/dcsb-* clones (KO7 and KO8) were identified.
